# Review on cardioprotective strategies in the setting of chemotherapy-induced cardiotoxicity

**DOI:** 10.1186/s40959-026-00480-4

**Published:** 2026-04-13

**Authors:** Vinh Dao, Shray Amin, Thirumala Kammaripalle, Kenneth Manning

**Affiliations:** 1 Department of Internal Medicine, Cape Fear Valley Medical Center, Fayetteville, NC USA; 2 Department of Hematology/Oncology, Cape Fear Valley Medical Center, Fayetteville, NC USA

**Keywords:** Chemotherapy-induced cardiotoxicity, Cardio-oncology, Dexrazoxane, Beta-blockers, ACE inhibitors, ARBs, MRAs, Statins, Global longitudinal strain, Exercise, Liposomal doxorubicin, SGLT2 inhibitors, GLP-1 RA

## Abstract

**Background:**

Chemotherapy-induced cardiotoxicity represents a major challenge in the care of cancer patients, particularly with anthracyclines, HER2-directed therapies, and other cytotoxic agents. As cancer survival improves, preserving cardiovascular health has become increasingly important.

**Objective:**

This review summarizes current evidence on pharmacologic, non-pharmacologic, and novel drug-delivery strategies for preventing and managing CIC, with a focus on optimizing left ventricular function.

**Methods:**

A comprehensive literature search of PubMed identified randomized controlled trials, observational studies, systematic reviews, and meta-analyses evaluating cardioprotective strategies in adults receiving cardiotoxic chemotherapy. Outcomes included left ventricular ejection fraction (LVEF), heart failure incidence, troponin levels, and global longitudinal strain (GLS).

**Results:**

Pharmacologic strategies with the strongest evidence include dexrazoxane and liposomal encapsulation of anthracyclines. Beta-blockers, ACE inhibitors, ARBs, MRAs, and statins to non-pharmacologic interventions like structured exercise programs and echocardiographic surveillance with GLS provide varying degrees of support. Still, the current literature is marked by heterogeneity in study design, endpoints, and patient populations. Other potential pharmacologic interventions include SGLT2 inhibitors and GLP-1 RA. While meta-analyses often show favorable trends, large-scale randomized controlled trials remain essential to validate these approaches, optimize patient selection, and standardize implementation. Until such data are available, clinicians should consider individualized risk assessment and adopt a multifaceted approach, incorporating both preventative and early detection strategies, to preserve cardiac function and maximize the safety of cancer therapy.

**Conclusion:**

A multifaceted approach incorporating pharmacologic therapy, exercise, imaging surveillance, and drug formulation strategies can potentially mitigate chemotherapy-induced cardiotoxicity. Large-scale, well-designed randomized trials are needed to optimize timing, patient selection, and combinations of cardioprotective interventions. Until such data are available, individualized risk assessment and early intervention remain key to preserving cardiac function in patients undergoing potentially cardiotoxic chemotherapy.

## Introduction

In 2022, the European Society of Cardiology presented guidelines on cardio-oncology to guide cancer therapy-related cardiovascular toxicity. Their definitions encompass a broad spectrum of pathology and include heart failure, myocarditis, coronary artery disease, peripheral vascular disease, arterial hypertension, and cardiac arrhythmias, along with the criteria for diagnosis of each [1]. These manifestations become paramount as new therapies, surgical advances, and earlier detection improve cancer outcomes. Of note, the incidence of cardiotoxicity varies depending on the type of chemotherapy given. Common chemotherapies implicated in cardiotoxicity include anthracyclines, HER2-directed therapies, alkylating agents, and immunotherapy.

Pharmacologic interventions such as angiotensin-converting enzyme inhibitors (ACEi), angiotensin receptor blockers (ARBs), beta-blockers, mineralocorticoid receptor antagonists (MRAs), statins, and dexrazoxane have shown varying degrees of efficacy in preserving cardiac function. On the other hand, non-pharmacologic approaches, including structured exercise programs and advanced echocardiographic techniques such as global longitudinal strain (GLS), offer additional avenues for early detection and prevention. Furthermore, novel drug delivery systems, such as liposomal encapsulation of anthracyclines, aim to reduce myocardial exposure to toxic agents while maintaining antitumor efficacy. This review attempts to summarize the current approaches to manage and prevent chemotherapy-induced cardiotoxicity with a focus on strategies to better optimize left ventricular dysfunction. By integrating insights from pharmacologic, imaging, lifestyle, and drug formulation perspectives, we aim to provide a comprehensive overview of current evidence and emerging approaches in the field of cardio-oncology.

## Methods

A comprehensive literature search was conducted to identify studies investigating cardioprotective strategies for chemotherapy-induced cardiotoxicity. We searched PubMed, Embase, and the Cochrane Library from using combinations of keywords related to chemotherapy-induced cardiotoxicity (e.g., “cardiotoxicity,” “chemotherapy,” “anthracyclines,” “trastuzumab”) and cardioprotective interventions (e.g., “dexrazoxane,” “beta blockers,” “ACE inhibitors,” “angiotensin receptor blockers,” “mineralocorticoid receptor antagonists,” “exercise,” “liposomal doxorubicin,” “global longitudinal strain”).

Eligible articles included randomized controlled trials (RCTs), observational studies, systematic reviews, and meta-analyses that assessed the efficacy of one or more cardioprotective strategies in adult patients undergoing chemotherapy with known cardiotoxic agents (e.g., anthracyclines, HER2-targeted therapies). Studies had to report on at least one cardiac outcome (e.g., left ventricular ejection fraction [LVEF], heart failure incidence, troponin levels, or global longitudinal strain [GLS]).

## Results

### Global longitudinal strain (GLS)

Currently, transthoracic echocardiography (TTE) and cardiovascular magnetic resonance (CMR) are the most common methods for evaluating left ventricular ejection fraction (LVEF). Cardiovascular magnetic resonance (CMR) is often limited due to its resource and cost restraints. Multiple-gated acquisition scans (MUGA) can also be done as a third line. Their key advantage lies in its high reproducibility and low variability. However, it lacks the ability to further characterize structural abnormalities and raises significant concerns about introducing additional radiation to the patient [2]. Given the additional radiation, the Eastern Association of Cardiovascular Imaging and the European Society of Cardiology recommend using three-dimensional (3D) TTE as initial imaging. Although the TTE can better evaluate the cardiac structure, it is still operator-dependent, leading to some variance [2].

Printezi et al. performed a cross-sectional study comparing MUGA LVEF to two-dimensional (2D) TTE, 3D TTE, and CMR. They analyzed 1017 patients and found that 3D TTE and harmonic TTE with contrast (2DHC) both correlated well with MUGA. The variability between them was within 10% LVEF units [2]. Walker et al. also conducted a study analyzing 50 women with HER2-positive breast cancer receiving trastuzumab after doxorubicin. In this study, serial MUGA, 2D TTE, 3D TTE, and CMR were performed at baseline, 6 months, and 12 months. They compared the left ventricular end diastolic volume of each of these modalities to CMR. It was found that a 2D TTE had a modest correlation with CMR (*r* = 0.69 at 12 months) while 3D TTE had a strong correlation (*r* = 0.91 at 12 months). MUGA also had a strong correlation (*r*= 0.90 at 12 months) [3]. Despite limited data, it seems that 3D TTE, 2D TTE with contrast, and CMR may be interchangeable with MUGA scans.

Efforts to mitigate cardiotoxicity from chemotherapy agents such as trastuzumab and anthracyclines have led to growing interest in Global Longitudinal Strain (GLS) as a potential tool to determine when to initiate some of the potentially cardioprotective agents discussed earlier for early detection. GLS, derived from echocardiographic imaging, assesses the longitudinal shortening of the left ventricle’s longitudinal fibers during systole as a negative percentage. Typical normal values range from − 18% to −22%. As the values become less negative and have a change of ≥ 15% from baseline, this indicates subclinical LV dysfunction and thus myocardial strain [4]. Compared to traditional transthoracic echocardiography (TTE), which focuses on left ventricular ejection fraction (LVEF), GLS has been theorized to detect subclinical cardiac dysfunction earlier [4].

The SUCCOUR trial attempted to answer the role of GLS by randomizing 331 anthracycline-treated patients to initiation of cardioprotective therapy triggered by a relative decrease in ≥ 12% GLS or > 10% LVEF. Patients were first started on ACEi/ARBs, followed by beta-blockers if there were any signs of cancer therapy-related cardiac dysfunction. The patients were followed to assess the changes in LVEF and the incidence of chemotherapy-induced cardiotoxicity. While no significant difference in LVEF change was observed between the GLS-guided group and the LVEF-monitored group at one year, the GLS group had a decreased incidence of chemotherapy-induced cardiotoxicity (5.8% vs. 13.7%; *p* = 0.02), and the 1-year LVEF was found to be 57 ± 6% versus 55 ± 7% (*p*= 0.05) [5]. At three-year follow-up, it was shown that more patients in the GLS-guided group were started on cardioprotective therapies. However, the change in LVEF was not statistically significant (−0.03% in the EF-guided arm compared to −0.02% in the GLS-guided arm [6].

The SUCCOUR-MRI trial evaluated using a worsening GLS as the criterion to start either metoprolol or bisoprolol and ramipril. If a patient was intolerant of ramipril, they were transitioned to either irbesartan or candesartan. In this study, there were 355 patients receiving anthracycline-based therapy with a baseline normal EF with at least one risk factor (diabetes, hypertension, dyslipidemia, smoking, or other cardiovascular disease) for chemotherapy-induced cardiotoxicity. The patients were randomized to obtaining usual care or initiating neurohormonal antagonists. With GLS and initiating cardioprotective agents, it was found that patients had less LVEF reduction compared to the usual care arm (−2.5 ± 5.4% vs. −5.6 ± 5.9%, *P*= 0.009) [7].

The PROTECT HER2 trial 110 patients receiving trastuzumab and/or pertuzumab-based regimens were divided to four arms: patients with normal LVEF and GLS, patients whose GLS decreased by > 15% from baseline, patients whose GLS decreased to < −15% who received prophylactic carvedilol, and patients whose GLS decreased to < −15% and did not receive carvedilol, and patients who developed cancer-therapy related cardiac dysfunction. This study showed no significant difference in GLS stability, development of chemotherapy-induced cardiotoxicity, or number of cancer therapy cycles completed [8].

Overall, the measurement of GLS has shown promise as an early diagnostic tool, consistently detecting subclinical dysfunction before LVEF changes. However, the implementation of cardioprotective therapies based on GLS findings has yielded only modest clinical benefits. Another challenge lies in the variability of the interventions across studies. SUCCOUR used beta-blockers and ACEi/ARBs, SUCCOUR-MRI used metoprolol and ramipril vs. irbesartan/candesartan, and PROTECT HER2 used carvedilol. Given these inconsistencies, the modest outcomes may reflect the limitations of current cardioprotective strategies rather than flaws in GLS itself. Furthermore, GLS is derived from 2D echocardiography, a modality inherently dependent on operator skill and patient characteristics. While clinical trials standardize techniques and equipment, real-world applications may face variability in image quality and interpretation. Body habitus, acoustic windows, and technician experience all affect reliability. Despite these limitations, such variability is not unique to GLS. Traditional TTE assessments of wall motion and LVEF are similarly affected. As GLS becomes more widely used, standardization and training will be essential. With ongoing research and the refinement of cardioprotective strategies, GLS may eventually play a larger role in managing patients receiving cardiotoxic chemotherapy. Until then, its integration into routine practice should be approached with measured optimism, careful cost-benefit analysis, and continued evaluation through high-quality randomized trials.

### Dexrazoxane

Dexrazoxane has been shown to provide cardioprotective effects and prevent chemotherapy-induced cardiotoxicity for patients with advanced or metastatic breast cancer receiving anthracycline-based therapy. It is thought to achieve this through its iron chelation properties, which decrease reactive oxygen species created from anthracyclines interacting with non-heme iron [9]. Furthermore, it is theorized that its ability to prevent the binding of anthracyclines to the Top2β-DNA complex and its inhibition of PAR poly (ADP-ribose) polymerase may improve cardiac function [10, 11]. However, it should be noted that the European Society of Cardiology recommends considering dexrazoxane in adult cancer patients with high and very high CTRCD risk where potential treatment requiring anthracycline-based therapy is necessary [1]. High-risk patients can be defined as those with established cardiac disease, prior anthracycline exposure, those who are planned to receive both anthracycline and radiation-based therapies, or patients with either a low-normal LVEF or mildly reduced LVEF.

Venturini et al. randomized 162 breast cancer patients to receive epirubicin therapy with or without dexrazoxane. The primary outcome was cardiac toxicity, defined as clinical signs of heart failure, a decrease in LVEF to ≤ 45%, or a decrease in baseline resting LVEF ≥ 20%. Cardiotoxicity was found to occur in 17 of the 78 patients (23.1%) in the epirubicin group without dexrazoxane, compared to 6 of 82 patients (7.3%) in the dexrazoxane group. The probability of developing cardiotoxicity was significantly lower in the dexrazoxane treatment group compared to the control (*P*= 0.006; odds ratio, 0.29; 95% confidence limit [CL], 0.09 to 0.78) [12].

Similar results have been found by Marty et al., who conducted a randomized 164 patients with breast cancer receiving anthracycline-based therapy to either dexrazoxane or placebo. The primary outcome was the incidence of cardiac events, which was defined as a 10% decrease in LVEF, a reduction in LVEF below 45%, or clinical signs of cardiac insufficiency based on the New York Heart Association of cardiac status. 10 patients in the dexrazoxane group (13%) experienced a cardiac event compared to 29 patients in the control group (39%). This indicated a relative risk of 68% (*P*< 0.001) [13].

Chow et al. also randomized 1308 pediatric patients with either acute lymphoblastic leukemia/lymphoma, Hodgkin lymphoma, or osteosarcoma receiving doxorubicin to dexrazoxane or placebo. The primary cardiovascular outcomes were hard cardiovascular endpoints defined as cardiomyopathy, ischemic heart disease, and stroke. The dexrazoxane group experienced a hard cardiovascular endpoint in 5 of the 90 patients compared to the control group who experienced it in 13 of 73 patients (*P*= 0.002) [14].

Meta-analyses have also shown dexrazoxane to have cardioprotective effects. Macedo et al. analyzed 7 randomized control trials and 2 retrospective trials with 2177 patients with breast cancer receiving anthracycline-based therapy. They found that the risk of clinical heart failure with dexrazoxane was significantly reduced (RR: 0.19; 95% CI: 0.09 to 0.40; *p*< 0.001) [15].

Collectively, the evidence supporting the cardioprotective effects of dexrazoxane is substantial. However, its clinical use remains limited by relatively narrow regulatory indications. In the United States, dexrazoxane is approved by the FDA for patients with metastatic breast cancer who have received a cumulative anthracycline dose of at least 300 mg/m² and who require continued anthracycline therapy. Several gaps in the evidence base have constrained broader adoption. These include limited data regarding its cardiac safety in patients with pre-existing left ventricular systolic dysfunction or established heart failure, a lack of randomized trial data in adults with leukemia and lymphoma, and incomplete characterization of its potential myelosuppressive effects [16]. This, in particular, becomes a concern in hematologic malignancies where bone marrow reserve may already be compromised. Additional RCTs in diverse adult oncology populations would help clarify these safety considerations and better define dexrazoxane’s role in treatment regimens.

### Beta blockers

As one of the core medications in guideline-directed medical therapy, beta-blockers remain a cornerstone in treating patients with heart failure with reduced ejection fraction. Their mechanism of action revolves around counteracting sympathetic activation, leading to a decrease in heart rate, reducing myocardial oxygen demand, and improving diastolic filling times. Given that chemotherapy-induced cardiotoxicity can lead to left ventricular dysfunction, many of the same processes for typical heart failure patients on beta blockers have been thought to apply to cancer patients, with many trials showing varying results.

The PRADA trial randomized 130 women with early breast cancer receiving anthracycline therapy in a 1:1:1:1 fashion to receive candesartan, metoprolol, candesartan and metoprolol, or placebo. The primary endpoint was a decrease in LVEF from baseline to follow-up. The placebo group had a decrease in LVEF from baseline at time of follow-up of −2.8% (95% CI − 4.3, − 1.3). When compared to the placebo group, the candesartan alone arm had a decrease in LVEF at time of follow-up of −0.9% [(95% CI − 2.3, 0.4); *P* = 0.025], the candesartan-metoprolol group had a LVEF decrease of −0.6% ((95% CI −2.1, 0.8); *P* = 0.075), and the metoprolol alone group had a decrease in LVEF of −2.5% [(95% CI −3.9, −1.1) *P*= 0.71) [17].

The MANTICORE trial evaluated trastuzumab-related cardiotoxicity for patients with HER2- positive breast cancer and had a 1:1:1 randomization to bisoprolol, perindopril, or placebo. The primary endpoint was the difference in change in left ventricular end diastolic volume (LVEDV) after 17 cycles of trastuzumab. This study found the mean change in LVEDV for the perindopril group LVEDV was + 7 ± 14 mL/m^2,^ while the bisoprolol was + 8 mL ± 9 mL/m^2^ and the placebo group was + 4 ± 11 mL/m^2^(*P* = 0.36). However, secondary analyses revealed that mean absolute LVEF decrease secondary to trastuzumab was mitigated with the bisoprolol group (−1 ± 5%) when compared to perindopril (−3 ± 4%) and placebo (−5 ± 5%) groups (*P*= 0.001) [18].

The CECCY trial randomized 200 patients with HER2-negative breast cancer who received 240 mg/m2 of anthracycline therapy to receive carvedilol or placebo. The primary endpoint was the prevention of ≥ 10% LVEF reduction after 6 months. The primary endpoint was reached in 14 of the 96 patients in the carvedilol group and was reached in 13 of the 96 patients for the placebo group (*P* = 1.0). Secondary analyses did demonstrate that there was a lower incidence of diastolic dysfunction in the carvedilol group (24 of 96) compared to the placebo group (32 of 96) (*P*= 0.039) [19].

The OVERCOME trial randomized 36 patients with newly diagnosed leukemia and 54 undergoing autologous hematopoietic stem cell transplantation without any LV systolic dysfunction to enalapril and carvedilol or a control group. The primary endpoint was the absolute change in LVEF. At 6 months, the absolute decrease in the enalapril and carvedilol group was − 0.17 (−2.24 to 1.90) compared to the placebo group − 3.28 (−5.49 to −1.07) (*P*= 0.04) [20].

The SAFE trial randomized 174 patients with breast cancer being treated with anthracycline-based chemotherapy to either bisoprolol, ramipril, both drugs, or placebo. The primary endpoint was a worsening myocardial function of ≥ 10%, LVEF, and global longitudinal strain (GLS). After 12 months of therapy, LVEF worsened by 4.4% in the placebo group, 3.0% in the ramipril group, 1.9% in the bisoprolol group, and 1.3% in the group receiving both bisoprolol and ramipril (*P*= 0.03) [21].

In each of these trials, there has been a low sample size. The CARDIOTOX trial attempts to address this. It has randomized 1018 patients undergoing anthracycline-based chemotherapy to receive carvedilol or placebo. In this study, the goal is to titrate carvedilol to 25 mg twice daily. The primary endpoint is LVEF reduction of at least 10% resulting in the EF decreasing to < 50%, cardiovascular death, myocardial infarction, heart failure exacerbation, or clinically significant arrhythmia. This study is still ongoing, but remains the largest trial to date dedicated to deciphering the role of beta-blockers in preventing cardiotoxicity [22].

He et al. conducted a meta-analysis of 10 randomized controlled trials, encompassing 875 patients, who had anthracycline-based therapy and subsequent cardiotoxicity. This study was designed to compare beta-blockers’ efficacy and analyze carvedilol, metoprolol, bisoprolol, nebivolol, and placebo. The meta-analysis showed improvement in LVEF reduction for carvedilol and placebo (RR = 0.51 95%CI (0.14, 0.88), *P* = 0.007). Bisoprolol and nebivolol also showed a improvement in LEVEF reduction compared to placebo (RR = 0.67 95%CI (0.23, 1.10), *P* = 0.002). There was no statistically significant difference noted between metoprolol and placebo (RR = 0.06, 95%CI (− 0.44, 0.57), *P* = 0.803). He et al. concluded that carvedilol was the superior beta-blocker in chemotherapy-induced cardiotoxicity [23]. Huang et al. also observed a lower incidence of clinically overt cardiotoxicity in their meta-analysis of 6 RCTs with 533 patients that studied prophylactic carvedilol. The primary outcome was LVEF after chemotherapy. It was found that there was no statistically significance between prophylactic carvedilol and placebo. The mean difference of LVEF between the two at end of treatment was 1.74 (95% CI, − 0.18 to 3.66; *P*= 0.08) [24]. Other meta-analyses have attempted to characterize the effect of beta-blockers and angiotensin antagonists in patients receiving higher doses of anthracyclines. Yun et al. found that the treatment group of beta-blockers and angiotensin antagonists had a higher post-chemotherapy LVEF compared to placebo. The mean difference estimate (95% CI) was 6.06% (0.54 to 11.58), *p* = 0.03. They also reported a trend that patients receiving higher cumulative doses of anthracyclines typically saw a larger benefit with treatment.

Overall, although some studies show promise in using beta-blockers to prevent chemotherapy-induced cardiotoxicity, other studies show conflicting data. However, it is important to note that not all beta-blockers are identical in their pharmacologic properties, which may account for some of the variance seem among the difference in LVEF outcomes across the trials. Combination therapy with ACE inhibitors may also provide additional benefit. Regardless, large-scale randomized trials are needed to further determine their value in clinical practice. In addition, careful selection of the most appropriate beta-blockers should be considered in these trials. However, it appears that initiating beta-blockers might be a reasonable approach for high-risk populations.

### Angiotensin-converting enzyme inhibitor, angiotensin II receptor blockers, and angiotensin receptor-neprilysin inhibitors

Angiotensin-Converting Enzyme Inhibitors (ACEi), Angiotensin II Receptor Blockers (ARBs), and Angiotensin Receptor-Neprilysin Inhibitors (ARNIs) function by affecting the renin-angiotensin-aldosterone system. The inhibition of this system leads to counteracting oxidative stress, endothelial dysfunction, and myocardial remodeling induced by chemotherapy [25].

The evidence concerning ACEi comes from 2 of the previously discussed trials, the OVERCOME and MANTICORE trials. The OVERCOME trial found that the absolute decrease in the enalapril and carvedilol group was − 0.17 (−2.24 to 1.90) compared to the placebo group − 3.28 (−5.49 to −1.07) (*P*= 0.04) [12]. On the other hand, the MANTICORE trial did not show any difference in the change of LVEDV between the perindopril arm and placebo. This study found the mean change in LVEDV for the perindopril group LVEDV was + 7 ± 14 mL/m^2,^ while the bisoprolol was + 8 mL ± 9 mL/m^2^ and the placebo group was + 4 ± 11 mL/m^2^(*P*= 0.36) [10].

Regarding the data on the usage of ARBs, the previously mentioned PRADA trial showed that candesartan has a significant short-term impact on LVEF reduction. The overall decline in LVEF was 2.6% (95% CI 1.5, 3.8) in the placebo group and 0.8% (95% CI −0.4, 1.9) in the candesartan group (*P* = 0.026) [9] However, in the extended follow-up of the PRADA trial, which had a 2-year follow-up, no significant long-term difference in LVEF was noted. With the extended follow-up, the overall decline in LVEF for placebo was − 1.8% (−3.0, − 0.6) compared to the candesartan group − 1.7% (−2.8, −0.5) (*P*= 0.91) [26].

Boekhout et al. randomized 210 women with early-stage HER2-positive breast cancer receiving trastuzumab in a 1:1 ratio to receive candesartan or placebo. These patients were monitored for 78 weeks, and the primary endpoint was a decline in LVEF of > 15% or an absolute value < 45%. They found that 20 patients in the candesartan arm met the primary endpoint compared to 16 in the placebo arm (95% CI, − 7% to 15%; *P*= 0.58) [27].

The PRADA II randomized 138 women who were planned to undergo either neoadjuvant and/or adjuvant anthracycline-based chemotherapy to receive sacubitril/valsartan or placebo in a 1:1 fashion. The patients had a 3 month and 18 month follow-up, where the primary outcome was change in LVEF by CMR from time of randomization to 18 months. The study found that the overall decline in LVEF in the sacubitril/valsartan group was 1.1% (95% CI, − 0.01 to 2.2) compared to the placebo group, which had an overall decline in LVEF of 2.2% (95% CI, 1.1 to 3.3). The difference between the groups was 1.1% (95% CI, − 0.4 to 2.7; *P*= 0.16) [28].

The SARAH trial randomized 114 men and women with elevated cardiac troponin I levels above the 99th percentile for their specific sex after any anthracycline chemotherapy session. They were randomized in a 1:1 ratio to receive either sacubitril-valsartan or placebo for 6 months. The primary outcome was a > 15% reduction in GLS at 6 months. The study found that the primary endpoint occurred in 4 patients in the sacubitril-valsartan group and 14 patients in the placebo grou (OR 0.23 [95% CI, 0.07–0.75]; *P =*0.015) [29].

Gao et al. performed a systematic review of 15 RCTs using an ACEi, ARB, or beta blocker to prevent chemotherapy-induced cardiotoxicity with a total of 1977 patients. The primary endpoint was LVEF. Of the 6 studies for ACEi/ARB, they found there was a significantly higher LVEF (χ2 = 136.62, I2 = 96.3%, *p* = 0.000; SMD 0.915, 95% CI 0.116 to 1.714). The same higher LVEF was found for the 13 studies containing beta blockers with a χ2 = 41.03, I2 = 70.8%, *p*= 0.000; SMD 0.393, 95% CI 0.177 to 0.610 [30].

Dong et al. conducted a meta-analysis of 5 RCTs containing 702 early-stage breast cancer patients receiving ACEi/ARB treatment. The primary endpoint was LVEF. It was found that there was a statistically significant change in mean LVEF with ACEi/ARB arms having a mean difference of 4.08% (95% CI: 0.8% to 7.35%, *P*= 0.01) compared to placebo [31].

Vaduganathan et al. performed a meta-analysis of 17 RCTs containing 1984 patients receiving potentially cardiotoxic chemotherapy as well as ACEi/ARB or beta blockers. The primary outcome was the change in LVEF by the end of each trial. This study grouped all medical interventions under neurohormonal therapies and found that the neurohormonal therapy arm had a significantly higher LVEF with a standardized mean difference of 1.04% (95% CI 0.57, 1.50 I^2^ = 96%, *p*< 0.001) [32].

In conclusion, the use of ACEi/ARBs/ARNIs in preventing and minimizing chemotherapy-induced cardiotoxicity has mixed evidence. Although many meta-analyses provide evidence for their usage, most RCTs have failed to provide conclusive evidence. However, these RCTs have had variations in patient populations, chemotherapy regimens, and study design. Similarly, ACE inhibitors and ARBs differ in their pharmacologic effects, and individual agents may vary in cardioprotective efficacy, which could partly explain the heterogeneous findings across trials. As such, large-scale, randomized controlled trials are essential to further define optimal timing, dosage, patient selection, and mechanism of action to maximize the cardioprotective benefits of ACEis and ARBs in this setting.

### Mineralocorticoid receptor antagonists

Mineralocorticoid receptor antagonists (MRAs), including spironolactone and eplerenone, have been a cornerstone in treating heart failure with reduced ejection fraction. By blocking the mineralocorticoid receptor through inhibiting aldosterone, MRAs reduce collagen deposition, oxidative injury, and endothelial dysfunction, leading to the preservation of myocardial structure and function. Given the mechanistic overlap between heart failure and anthracycline-induced myocardial injury, MRAs have garnered attention as potential cardioprotective agents in oncology.

Akpek et al. conducted a randomized controlled trial of 83 breast cancer patients receiving anthracycline-based chemotherapy, assigning 43 patients to receive spironolactone and 40 to a control group. The primary outcome was change in left ventricular ejection fraction (LVEF). After chemotherapy, the spironolactone group experienced a modest decline in LVEF from 67.0 ± 6.1% to 65.7 ± 7.4% (*P* > 0.05), while the control group showed a significantly greater reduction from 67.7 ± 6.3% to 53.6 ± 6.8% (*P* < 0.001). The between-group difference in LVEF decline was statistically significant (*P*< 0.001), suggesting that spironolactone may offer cardioprotection against anthracycline-induced toxicity [33].

In the ELEVATE trial, Davis et al. randomized 44 breast cancer patients undergoing anthracycline-based chemotherapy to receive either eplerenone or placebo. The primary endpoint was the average early diastolic tissue velocity of the mitral annulus (E′) at 6 months. At follow-up, there was no statistically significant difference in E′ between the eplerenone and placebo groups (mean difference: 0.03 cm/s; 95% CI: − 0.55 to 0.61; *P*= 0.92), indicating that eplerenone did not provide measurable cardioprotective benefit in this cohort [34].

While early findings from Akpek et al. suggest that spironolactone may attenuate anthracycline-induced declines in LVEF, the ELEVATE trial’s neutral results with eplerenone highlight the variability in cardioprotective outcomes among MRAs. Differences in drug pharmacodynamics, patient populations, or chosen cardiac endpoints may account for the divergent results [33]. These mixed findings underscore the need for larger, well-powered trials to clarify the role of MRAs in preventing chemotherapy-related cardiotoxicity and to determine whether specific agents within this class offer superior protection. Nonetheless, MRAs remain a promising avenue for cardioprotection and merit further investigation.

### Statins

Statins have long been established as cardioprotective agents in various cardiovascular diseases such as coronary artery disease, diabetes, and peripheral vascular disease. Given the well-documented cardiotoxicity of certain chemotherapy agents, efforts have been made to determine whether statins can confer similar cardioprotective benefits in patients undergoing cancer treatment.

The STOP-CA trial randomized 300 patients with lymphoma to receive either atorvastatin 40 mg daily or placebo for 12 months, beginning before initiation of anthracycline-based chemotherapy. The primary endpoint was the proportion of patients experiencing a ≥ 10% decline in left ventricular ejection fraction (LVEF) to < 55%. This occurred in 9% of patients in the atorvastatin group compared to 22% in the placebo group (absolute difference: − 13%; 95% CI: − 23.0 to − 3.9; *P* = 0.002). A secondary endpoint, an absolute decline in LVEF ≥ 5%, was also significantly less frequent in the statin group (13% vs. 29%; absolute difference: − 16%; 95% CI: − 26.7 to − 5.4; *P*= 0.003). These findings suggest that high-intensity statin therapy confers cardioprotective effects during anthracycline treatment [35].

Kettana et al. conducted a randomized controlled trial of 50 patients with HER2-positive breast cancer receiving trastuzumab-based therapy, assigning participants to rosuvastatin or placebo for 6 months. The primary outcome was change in left ventricular ejection fraction (LVEF) at 3 and 6 months. At 6 months, the rosuvastatin group had a mean LVEF decline of − 3.44%, compared to − 8.22% in the placebo group (*P* = 0.001). At 3 months, LVEF declined by − 2.68% in the statin group versus − 6.72% in the placebo group (*P*= 0.015), reinforcing rosuvastatin’s potential to mitigate trastuzumab-associated cardiotoxicity [36].

Mohamad et al. conducted a randomized controlled trial of 110 breast cancer patients scheduled to receive anthracycline-based chemotherapy, assigning participants to either atorvastatin 40 mg daily or placebo. The primary endpoint was the change in left ventricular ejection fraction (LVEF) at 6 months. The placebo group experienced a greater mean decline in LVEF (–7.06%) compared to the atorvastatin group (–3.64%; *P* < 0.01). Additionally, the incidence of chemotherapy-induced cardiotoxicity, defined as a reduction in LVEF of > 10% to a value < 53%, was significantly lower in the statin group (7.3%) than in the placebo group (22.2%; *P*= 0.02). These findings support a cardioprotective role for atorvastatin in patients receiving anthracycline therapy [37].

Thavendiranathan et al. conducted a randomized controlled trial of 112 patients at increased risk for anthracycline-related cardiac dysfunction, assigning them to receive atorvastatin 40 mg daily or placebo. The primary endpoint was left ventricular ejection fraction (LVEF) measured by cardiac magnetic resonance imaging approximately 22 days after the final anthracycline dose. Post-treatment LVEF was 58.1% in the statin group and 58.3% in the placebo group (mean difference: − 0.2%; 95% CI: − 2.3 to 1.8; *P*= 0.86), indicating no significant cardioprotective effect of atorvastatin in this cohort [38].

The current body of evidence generally supports the hypothesis that statins may offer cardioprotective benefits in patients undergoing chemotherapy, particularly those treated with anthracyclines. However, inconsistencies across trials and variations in patient populations, statin types, and chemotherapy regimens limit the strength of these conclusions. An important limitation in many studies is the lack of control for pre-existing cardiovascular comorbidities. In conclusion, statin therapy may offer cardioprotective effects in certain subgroups of chemotherapy patients, particularly those with existing cardiovascular risk factors. However, further research—particularly well-designed, large-scale RCTs—is needed to determine whether statin therapy should be universally recommended in the setting of cancer treatment.

### Exercise

While pharmacologic interventions have shown varying degrees of promise, non-pharmacologic approaches, such as structured exercise programs, are emerging as potential cardioprotective tools. Exercise has been shown to positively influence cardiac remodeling, vascular function, and oxidative stress, which is theorized to decrease chemotherapy-induced cardiotoxicity.

Diaz-Balboa et al. conducted a RCT with 122 early-stage breast cancer patients receiving anthracyclines and/or anti-HER2 therapy. Patients were randomized to a 6-month supervised cardio-oncology rehabilitation (CORe) program or usual care with general exercise advice. The primary endpoint was examining the effectiveness of the CORe program in preventing a LVEF drop of ≥ 10% to a value < 53% or a decrease > 15% in global longitudinal strain (GLS). The CORe group exhibited significantly less decline in left ventricular ejection fraction (LVEF) (−1.5%, *p* = 0.006) and reduced body mass index (BMI) in obese patients (3.1 kg/m^2^, *p*= 0.037), indicating that supervised exercise can mitigate chemotherapy-related cardiotoxicity and support weight management [39].

Schneider et al. conducted a randomized controlled trial of 57 patients with breast cancer or lymphoma receiving anthracycline-based chemotherapy, comparing supervised exercise during treatment to delayed exercise post-treatment. Both groups received activity trackers. There was no significant difference in the primary outcome of global longitudinal strain (GLS) at chemotherapy completion (mean change: − 7.4% vs. − 6.2%; *P* > 0.05), nor in secondary outcomes such as peak VO₂ (–1.0 vs. − 1.1 mL/kg/min) or median daily moderate-to-vigorous physical activity (33 vs. 32 min; *P* > 0.05). However, in pooled analysis, each additional 10 min of MVPA per day was associated with a 1.11% smaller decline in GLS (β = − 1.11; *P* = 0.001) and a 0.55 ng/L smaller increase in high-sensitivity troponin T (β = − 0.55; *P*= 0.001), suggesting that overall activity level may mitigate cardiotoxicity [40].

Ansund et al. compared resistance training + high-intensity interval training (RT-HIIT) vs. aerobic training + HIIT (AT-HIIT) vs. usual care during chemotherapy in breast cancer patients. They randomized 74 women to RT-HIIT, 72 women to AT-HIIT, and 60 women to usual care. The primary outcomes were the effects on cardiac biomarkers (e.g., NT-proBNP) and cardiac function post-treatment. One-year post-baseline, both exercise groups showed significantly lower levels of NT-proBNP compared to controls (*p* = 0.01), indicating a potential long-term cardioprotective effect of high-intensity exercise during chemotherapy. Additionally, the interventions were safe and well-tolerated.

Exercise represents a promising and accessible intervention to counteract the cardiotoxic effects of chemotherapy. While preliminary studies are encouraging, further large-scale, randomized trials are needed to define optimal exercise regimens, timing, and patient selection. Nonetheless, integrating exercise into cancer care holds significant potential to improve both cardiac outcomes and overall survivorship in patients at risk of chemotherapy-induced cardiotoxicity.

### Liposomal encapsulation of anthracyclines

Anthracyclines are limited by dose-dependent and cumulative cardiotoxicity, which can lead to irreversible cardiac dysfunction. In response to this challenge, liposomal encapsulation of doxorubicin, particularly pegylated liposomal doxorubicin (PLD), has emerged as a potential strategy to mitigate these adverse effects while preserving antitumor efficacy. Multiple randomized controlled trials and meta-analyses have evaluated the safety and efficacy of liposomal formulations compared to conventional doxorubicin. These studies collectively suggest that liposomal doxorubicin significantly reduces the risk of cardiotoxicity without compromising therapeutic response, offering a promising alternative for patients at elevated cardiac risk.

Xing et al. conducted a meta-analysis of ten randomized controlled trials including 2,889 patients with advanced breast cancer, comparing liposomal doxorubicin-based chemotherapy to conventional doxorubicin. Liposomal formulations were associated with a significantly lower risk of cardiotoxicity (OR = 0.46; 95% CI: 0.23 to 0.91; *P* = 0.03) and a higher overall response rate (OR = 1.25; 95% CI: 1.02 to 1.54; *P*= 0.03), indicating improved cardiac safety without compromising therapeutic efficacy. These findings support liposomal encapsulation as a safer alternative for patients at risk of anthracycline-induced cardiotoxicity [41].

Smith et al. conducted a systematic review and meta-analysis of 55 randomized controlled trials examining cardiotoxicity across various anthracycline regimens. Compared to conventional doxorubicin, liposomal doxorubicin significantly reduced the risk of clinical cardiotoxicity (OR = 0.18; 95% CI: 0.08–0.38; *P* < 0.0001; *I²* = 0%), subclinical cardiotoxicity (RR = 0.31; 95% CI: 0.20–0.48; *P* < 0.0001; *I²* = 48.5%), and any cardiotoxic event, including both clinical and subclinical outcomes (RR = 0.30; 95% CI: 0.21–0.43; *P* < 0.0001; *I²* = 32.2%). No cases of congestive heart failure were reported in comparisons between liposomal doxorubicin and epirubicin, and there was no significant difference in overall cardiotoxicity (RR = 1.15; 95% CI: 0.47–2.84; *P*= 0.754). These findings support the use of liposomal formulations as a cardioprotective strategy during anthracycline therapy [42].

O’Brien performed a Phase III RCT to evaluate pegylated liposomal doxorubicin (PLD) with conventional doxorubicin as first-line treatment in 509 patients with metastatic breast cancer. Cardiotoxicity was measured through a reduction in LVEF. Cardiotoxicity with conventional doxorubicin compared to PLD had a hazard ratio of 3.16, *p*< 0.001 [43]. These findings demonstrate that PLD maintains anticancer activity with an improved safety profile, making it a favorable option for patients requiring anthracycline-based chemotherapy.

Rafiyath et al. conducted a meta-analysis to assess the safety and toxicity profiles between liposomal and conventional doxorubicin. 9 RCTs were included with 2220 patients analyzed. 1112 patients received liposomal formulations while 1108 received conventional doxorubicin. Patients receiving the liposomal formulation experienced significantly fewer cardiotoxic events (7.4% vs. 26%; *p*= 0.03). Liposomal formulations also had a lower incidence of heart failure with an odds ratio of 0.34. The study concluded that liposomal doxorubicin has a more favorable toxicity profile, particularly in terms of cardiac safety [44]. These findings reinforce the value of liposomal encapsulation in minimizing adverse effects without compromising anticancer efficacy.

The accumulated evidence from randomized trials and meta-analyses strongly supports the use of liposomal doxorubicin as a safer and equally effective alternative to conventional anthracycline therapy. Studies consistently demonstrate a marked reduction in cardiotoxic events, including reduced incidence of heart failure and significant preservation of left ventricular function, when liposomal formulations are used. Importantly, these benefits are achieved without sacrificing overall response rates or clinical efficacy. As such, liposomal doxorubicin can be considered a reasonable alternative to standard doxorubicin for patients who would benefit from anthracycline-including chemotherapy but who are at increased risk of anthracycline-induced cardiotoxicity, aligning oncologic outcomes with improved cardiovascular safety.

### Potential pharmacologic interventions

Beyond previously discussed strategies, several emerging pharmacologic interventions are being explored for potential cardioprotective effects during chemotherapy, including sodium–glucose cotransporter 2 inhibitors (SGLT2) and glucagon-like peptide-1 receptor agonists (GLP-1 RAs). SGLT2 inhibitors have demonstrated clear benefits in heart failure populations unrelated to cancer therapy, including reductions in hospitalization and cardiovascular mortality. GLP-1 receptor agonists, while not consistently associated with heart failure prevention, have shown potential in some analyses to reduce major adverse cardiovascular events. Importantly, the evidence supporting both classes in the context of chemotherapy-induced cardiotoxicity remains limited, consisting largely of retrospective observational studies. These findings highlight a promising area of investigation, with prospective randomized trials currently underway or needed to establish efficacy and safety in oncology populations.

Some emerging retrospective evidence for SGLT2 inhibitor was seen in retrospective cohort analysis done by Avula et al. They identified patients with type 2 diabetes mellitus who were receiving active treatment consisting of anthracyclines, alkylating agents, antimetabolites, monoclonal antibodies, small-molecule tyrosine kinase inhibitors, or proteasome inhibitors. There were 1280 patients in this study. The primary endpoint was heart failure exacerbations and all-cause mortality over 2 years. They found that patients who were receiving an SGLT2 inhibitor had a lower risk for a heart failure exacerbation (OR: 0.483 [95% CI: 0.36–0.65]; *P* < 0.001) and all-cause mortality (OR: 0.296 [95% CI: 0.22–0.40]; *P*= 0.001) [45].

Mohsin et al. performed a meta-analysis for 2817 patients who were receiving SGLT2 inhibitors and had received anthracycline-based treatment or doxorubicin. The primary endpoints were overall mortality and cardiovascular events defined as heart failure hospitalization or heart failure incidence. The mean follow-up was 1.6 years. The study found that there was a reduction in overall mortality (RR = 0.52 (0.33–0.82); *p* = 0.005) and heart failure hospitalization (RR = 0.20 (0.04–1.02); *p* = 0.05). However, there was no significant reduction in heart failure incidence (RR = 0.50 (0.20–1.16); *p*= 0.11) [46].

Bhatti et al. performed a retrospective analysis of 95,203 patients with type 2 diabetes mellitus and cancer and without any history of heart failure. These patients received one of the following therapies: anthracyclines, alkylating agents, antimetabolites, monoclonal antibodies, small-molecule tyrosine kinase inhibitors, and proteasome inhibitors. Of these patients, only 9403 were on an SGLT inhibitor. After propensity-score matching, 8,675 patients were included in each cohort. The primary endpoint was cancer therapy-related cardiac dysfunction defined as new onset cardiomyopathy, heart failure, or requiring intravenous loop diuretics within 12 months of the index event. The study found that the SGLT2 inhibitor cohort had a lower risk of developing cancer therapy-related cardiac dysfunction (HR: 0.76: 95% CI: 0.69–0.84, *P <* 0.001). The subgroup analysis also showed that the SGLT2 inhibitor cohort had reduced heart failure exacerbations (HR: 0.81; 95% CI: 0.72–0.90, *P <* 0.001), all-cause mortality (HR: 0.67; 95% CI: 0.61–0.74, *P <* 0.001), and all-cause hospitalizations/emergency department visits (HR: 0.93; 95% CI: 0.89–0.97, *P <*0.001) [47].

With regards to GLP-1 RA, Vignarajah et al. conducted a retrospective cohort study that examined 4946 patients with a history of cancer and that were receiving one of the following therapies: anthracyclines, alkylating agents, antimetabolites, monoclonal antibodies, small-molecule tyrosine kinase inhibitors, and proteasome inhibitors. After propensity-score matching, 837 patients remained in the GLP-1 RA cohort and 837 remained on the control group. The GLP-1 RA group was found to have a significant decrease in all-cause mortality (HR 0.57 [95% CI, 0.43–0.77]; *P* < 0.001), heart failure exacerbation (HR, 0.69 [95% CI, 0.56–0.85]; *P* < 0.001), and all-cause hospitalization (HR, 0.83 [95% CI, 0.72–0.95]; *P*< 0.001) [48].

However, there were several limitations with this study. The study was retrospective and observational, introducing the potential for residual confounding and selection bias despite propensity-score matching. Cancer types, treatment regimens, and baseline cardiovascular risk were heterogeneous, limiting generalizability. Therefore, while these results are supportive for a potential cardioprotective role for GLP-1 RAs, prospective randomized controlled trials are needed to establish efficacy, optimal timing, and safety in this population.

## Conclusion

Chemotherapy-induced cardiotoxicity remains a significant challenge in the care of cancer patients. Many breast cancer and lymphoma patients would ideally need to be treated with chemotherapies that can compromise long-term cardiac function. As survival improves due to advances in oncologic care, protecting cardiovascular health has become increasingly important. The current body of evidence provides varying degrees of support for the use of various cardioprotective strategies, ranging from pharmacologic agents such as dexrazoxane, beta-blockers, ACE inhibitors, ARBs, MRAs, and statins to non-pharmacologic interventions like structured exercise programs and echocardiographic surveillance with GLS. Furthermore, innovations in drug formulation, particularly liposomal encapsulation of anthracyclines, offer meaningful reductions in cardiotoxic risk without sacrificing efficacy.

However, despite these promising strategies, the current literature is marked by heterogeneity in study design, endpoints, and patient populations. Moreover, mechanistic differences between individual agents within cardioprotective drug classes may further influence clinical outcomes, potentially explaining some of the variability seen across trials and highlighting the importance of careful drug selection in future studies. While meta-analyses often show favorable trends, large-scale randomized controlled trials remain essential to validate these approaches, optimize patient selection, and standardize implementation.

The next several cases attempt to illustrate potential uses based on our findings. For example, a patient with HER2-positive breast cancer scheduled to receive trastuzumab after anthracycline therapy could undergo baseline GLS assessment. If GLS declines ≥ 12%, early initiation of a beta-blocker or ACE inhibitor may prevent further LV dysfunction, as suggested by the SUCCOUR trial. In another scenario, a high-risk patient receiving cumulative anthracyclines could be treated with pegylated liposomal doxorubicin to minimize cardiotoxic risk, potentially in combination with supervised exercise programs to support cardiac function further. Furthermore, if this patient had metastatic breast cancer and had high cumulative anthracycline exposure (e.g., ≥ 300 mg/m² of doxorubicin), they may benefit from the addition of dexrazoxane to reduce the risk of cardiotoxicity while allowing continuation of effective chemotherapy. Similarly, patients with pre-existing cardiovascular risk factors may benefit from statin therapy to mitigate chemotherapy-induced LVEF decline.

Until more definitive data are available, clinicians should continue to individualize risk assessment and adopt a multifaceted approach, integrating pharmacologic and non-pharmacologic strategies alongside early detection, to preserve cardiac function and maximize the safety of cancer therapy. Table 1 summarizes our findings and details our recommendations for each cardioprotective strategy.

**Table 1:** Cardioprotective Strategies for Cancer Therapy–Related Cardiotoxicity: Mechanisms, Evidence, and Clinical Guidance

Agent/Therapy	Mechanism	Strength/Evidence	Supporting Evidence from Article	Clinical Practice Suggestion
Global Longitudinal Strain (GLS)	Detects subclinical LV dysfunction via longitudinal left ventricle’s fibers during systole; detects subclinical myocardial strain earlier than LVEF.	Moderate but Mixed (RCTs: SUCCOUR, SUCCOUR-MRI)	GLS-guided initiation of cardioprotective therapy decreased the incidence of chemo-induced cardiotoxicity (5.8% vs. 13.7%; *p* = 0.02) and resulted in less LVEF reduction (−2.5% vs. −5.6%; *P*=0.009).	Incorporate baseline and serial GLS in moderate/high-risk patients. A relative reduction ≥ 15% from baseline should prompt consideration of cardioprotective therapy.
Dexrazoxane	Iron chelation (decreases reactive oxygen species); prevents anthracycline binding to the Top2β-DNA complex; inhibits PARP.	Strong (RCTs, Meta-analyses)	Significantly lowered cardiotoxicity probability (7.3% vs. 23.1%; *P*=0.006) and cardiac events (13% vs. 39%; *P* < 0.001). Risk of clinical heart failure significantly reduced (RR: 0.19).	Consider for adult patients at high risk of anthracycline-related cardiotoxicity, particularly those approaching cumulative doses ≥ 300 mg/m² (FDA-approved in metastatic breast cancer). Needs additional studies for indications outside of breast cancer
Beta-Blockers	Counteracts sympathetic activation; decreases heart rate, reduces myocardial O₂ demand, and improves diastolic filling times.	Moderate but Mixed (Multiple RCTs, Meta-analyses)	Bisoprolol mitigated absolute LVEF decrease (−1% vs. −5%; *P* = 0.001). Carvedilol lowered diastolic dysfunction incidence (*P* = 0.039). Carvedilol/enalapril combined had lower absolute LVEF decrease.	Reasonable in high-risk patients or those with early LVEF/GLS decline. Agent selection may influence outcomes; not all beta-blockers appear equivalent.
ACEi/ARBs/ARNIs	Inhibits the renin-angiotensin-aldosterone system; counteracts oxidative stress, endothelial dysfunction, and myocardial remodeling.	Moderate but Mixed (RCTs, Meta-analyses)	Candesartan mitigated short-term LVEF decline. Sacubitril-valsartan (ARNI) significantly reduced the incidence of > 15% GLS reduction in patients with elevated troponin (OR 0.23; *P* = 0.015).	Consider utilizing to counteract myocardial remodeling, particularly for patients demonstrating early signs of subclinical LV dysfunction or those with elevated cardiac biomarkers post-chemotherapy. Evidence appears to be heterogeneous across agents.
Mineralocorticoid Receptor Antagonists	Aldosterone blockade; reduces collagen deposition, oxidative injury, and endothelial dysfunction.	Low (Small RCTs)	Spironolactone attenuated LVEF decline post-chemotherapy (65.7% vs. 53.6% in control; *P* < 0.001). Eplerenone did not show significant cardioprotective benefit.	Insufficient evidence for routine prophylaxis. Potential differences between individual MRAs warrant further study.
Statins	Conveys pleiotropic cardioprotective effects established in various cardiovascular diseases.	Moderate (RCTs; additional large-scale trials needed)	Atorvastatin reduced the proportion of patients with ≥ 10% LVEF decline (9% vs. 22%; *P* = 0.002). Rosuvastatin mitigated mean LVEF decline at 6 months (−3.44% vs. −8.22%; *P* = 0.001).	Consider particularly in patients with existing ASCVD risk or statin indication; may provide adjunctive benefit during anthracycline therapy
Structured Exercise	Positively influences cardiac remodeling, vascular function, and oxidative stress.	Moderate (RCTs; additional large-scale trials needed)	Supervised cardio-oncology rehabilitation resulted in significantly less LVEF decline (−1.5%; *p* = 0.006). High-intensity exercise significantly lowered long-term NT-proBNP levels post-treatment.	Encourage supervised aerobic and resistance training into standard cancer care to positively influence cardiac remodeling and reduce overall cardiotoxicity when clinically feasible
Liposomal Anthracyclines	Liposomal encapsulation reduces myocardial tissue exposure to toxic agents while preserving antitumor efficacy.	Strong (Phase III RCT, Meta-analyses)	Lowered risk of cardiotoxicity (OR = 0.46) and significantly fewer cardiotoxic events (7.4% vs. 26%; *P* = 0.03) without compromising overall tumor response rates.	Utilize as a safer alternative to conventional doxorubicin for patients requiring anthracycline-inclusive regimens who are at elevated risk for cardiotoxicity.
Emerging Pharmacotherapies (SGLT2i & GLP-1 RAs)	Provides generalized cardioprotection; associated with reductions in heart failure exacerbations and cardiovascular mortality.	Early clinical evidence but positive (Retrospective Cohorts, Meta-analyses; additional large-scale trials needed)	Retrospective data show SGLT2 inhibitors reduce the risk of cancer therapy-related cardiac dysfunction (HR 0.76) and HF exacerbations. GLP-1 RAs associated with decreased all-cause mortality and HF exacerbations.	While retrospective data is promising for reducing heart failure events and mortality, prospective RCTs are required before incorporating these agents into standard cardioprotective protocols.

## Data Availability

All data used in this review are from previously published sources, which are properly cited in the manuscript.
